# Outbreak of Invasive Wound Mucormycosis in a Burn Unit Due to Multiple Strains of Mucor circinelloides f. circinelloides Resolved by Whole-Genome Sequencing

**DOI:** 10.1128/mBio.00573-18

**Published:** 2018-04-24

**Authors:** Dea Garcia-Hermoso, Alexis Criscuolo, Soo Chan Lee, Matthieu Legrand, Marc Chaouat, Blandine Denis, Matthieu Lafaurie, Martine Rouveau, Charles Soler, Jean-Vivien Schaal, Maurice Mimoun, Alexandre Mebazaa, Joseph Heitman, Françoise Dromer, Sylvain Brisse, Stéphane Bretagne, Alexandre Alanio

**Affiliations:** a Institut Pasteur, National Reference Center for Invasive Mycoses and Antifungals (NRCMA), Molecular Mycology Unit, Paris, France; b CNRS UMR2000, Paris, France; c Institut Pasteur–Bioinformatics and Biostatistics Hub–C3BI, USR 3756 IP CNRS–Paris, France; d South Texas Center for Emerging Infectious Diseases (STCEID), Department of Biology, the University of Texas at San Antonio, San Antonio, Texas, USA; e UMR 942, INSERM, Service d’Anesthésie-Réanimation, Assistance Publique-Hôpitaux de Paris (AP-HP), Groupe Hospitalier Saint-Louis-Lariboisière-Fernand-Widal, Paris, France; f Université Paris Diderot, Sorbonne Paris Cité, Paris, France; g Service de Chirurgie Plastique, Reconstructrice, Esthétique et Traitement Chirurgical des Brûlés, Assistance Publique-Hôpitaux de Paris (AP-HP), Groupe Hospitalier Saint-Louis-Lariboisière-Fernand-Widal, Paris, France; h Service de Maladies Infectieuses et Tropicales, Assistance Publique-Hôpitaux de Paris (AP-HP), Groupe Hospitalier Saint-Louis-Lariboisière-Fernand-Widal, Paris, France; i Laboratoire de Microbiologie, Hôpital Saint-Louis, Assistance Publique-Hôpitaux de Paris (AP-HP), Groupe Hospitalier Saint-Louis-Lariboisière-Fernand-Widal, Paris, France; j Service de Biologie Médicale, Hôpital d’Instruction des Armées, Clamart, France; k Centre de Traitement des Brulés, Hôpital d’Instruction des Armées, Clamart, France; l Molecular Genetics and Microbiology, Duke University Medical Center, Durham, North Carolina, USA; m Institut Pasteur, Biodiversity and Epidemiology of Bacterial Pathogens, Paris, France; n Laboratoire de Parasitologie-Mycologie, Hôpital Saint-Louis, Assistance Publique-Hôpitaux de Paris (AP-HP), Groupe Hospitalier Saint-Louis-Lariboisière-Fernand-Widal, Paris, France; Vallabhbhai Patel Chest Institute

**Keywords:** Mucor circinelloides, Mucorales, mucormycosis, strains, burn, clade, genome, genotype, mixed infection, outbreak, whole-genome sequencing, wound

## Abstract

Mucorales are ubiquitous environmental molds responsible for mucormycosis in diabetic, immunocompromised, and severely burned patients. Small outbreaks of invasive wound mucormycosis (IWM) have already been reported in burn units without extensive microbiological investigations. We faced an outbreak of IWM in our center and investigated the clinical isolates with whole-genome sequencing (WGS) analysis. We analyzed M. circinelloides isolates from patients in our burn unit (BU1, Hôpital Saint-Louis, Paris, France) together with nonoutbreak isolates from Burn Unit 2 (BU2, Paris area) and from France over a 2-year period (2013 to 2015). A total of 21 isolates, including 14 isolates from six BU1 patients, were analyzed by whole-genome sequencing (WGS). Phylogenetic classification based on *de novo* assembly and assembly free approaches showed that the clinical isolates clustered in four highly divergent clades. Clade 1 contained at least one of the strains from the six epidemiologically linked BU1 patients. The clinical isolates were specific to each patient. Two patients were infected with more than two strains from different clades, suggesting that an environmental reservoir of clonally unrelated isolates was the source of contamination. Only two patients from BU1 shared one strain, which could correspond to direct transmission or contamination with the same environmental source. In conclusion, WGS of several isolates per patients coupled with precise epidemiological data revealed a complex situation combining potential cross-transmission between patients and multiple contaminations with a heterogeneous pool of strains from a cryptic environmental reservoir.

## IMPORTANCE

Invasive wound mucormycosis (IWM) is a severe infection due to environmental molds belonging to the order Mucorales. Severely burned patients are particularly at risk for IWM. Here, we used whole-genome sequencing (WGS) analysis to resolve an outbreak of IWM due to Mucor circinelloides that occurred in our hospital (BU1). We sequenced 21 clinical isolates, including 14 from BU1 and 7 unrelated isolates, and compared them to the reference genome (1006PhL). This analysis revealed that the outbreak was mainly due to multiple strains that seemed patient specific, suggesting that the patients were more likely infected from a pool of diverse strains from the environment rather than from direct transmission among them. This study revealed the complexity of a Mucorales outbreak in the settings of IWM in burn patients, which has been highlighted based on WGS combined with careful sampling.

## INTRODUCTION

Mucormycosis is a rare and life-threatening infection caused by Mucorales belonging to the subphylum *Mucoromycotina* (1). These molds are ubiquitously distributed in the environment and mostly disseminated through airborne spores, which can be considered infective propagules responsible mainly for respiratory (lung and sinuses), wound, and skin infections (2).

Patients at risk for mucormycosis are immunocompromised (hematological malignancies, hematopoietic stem cell transplantation, solid organ transplant, and steroid therapy) or have diabetes mellitus, deferoxamine treatment, trauma, or severe burns (2–4). Among skin-related infections, contaminated materials (Elastoplast bandages, tape, tongue depressors, ostomy bags, and linens) have been implicated as the causes of local or disseminated infections in patients with various underlying diseases (5). Specifically, in burn patients, invasive wound mucormycosis (IWM) has been reported in both small series (6) and epidemiological surveys (7–10).

Over the past 10 years, outbreaks of mucormycosis have been increasingly reported in various environments. In humans, nosocomial outbreak cases occurring before 2008 were reviewed by Antoniadou in 2009 (11). Antoniadou found 12 reported outbreaks and two pseudoepidemics of cases since 1977. Mucormycosis outbreaks have been reported in the United States, United Kingdom, and Europe (11). Since 2008, outbreaks or clustered cases have been reported after the tornadoes in Joplin, MO (13 patients) (12, 13), in an intensive care unit (ICU) in France (3 patients) (14), in adults (6 patients) (15) or infants (5 patients) (16) exposed to contaminated linens in the United States, in infants in Egypt (5 patients) (17), and in patients undergoing arthroscopy in Argentina (40 patients) (18). More specifically, in a Belgian burn unit, Christiaens et al. described an outbreak of Lichtheimia corymbifera associated with nonsterile Elastoplast bandage contamination in seven burn patients, including five with infection and two with colonization (19). In this study, the authors did not have evidence for the genotypic relatedness of the strains between patients and material strains. In addition, a large outbreak due to yogurt contamination in the United States responsible for digestive symptoms (nausea, cramps, vomiting, and diarrhea) in about 300 individuals has been described recently (20). In this study, phylogenetic analysis and whole-genome sequence (WGS) analysis yielded new information on the genetic structure of Mucor circinelloides. Mucor circinelloides is a single species consisting of four different formae (f. circinelloides, f. griseocyanus, f. janssenii, and f. lusitanicus), with forma circinelloides the most commonly involved in human mucormycosis (20, 21).

Between 2013 and 2015, we faced an outbreak of proven IWM due to M. circinelloides f. circinelloides in a burn unit (BU) in Hôpital Saint-Louis (SLS), Paris, France, involving six patients raising the hypothesis of a common source of contamination. The outbreak was not suspected until M. circinelloides was recovered from wounds of patient P04. Over the same period of time, 4 additional cases that occurred in a burn unit (Burn Unit 2 [BU2]) of another hospital in a Paris suburb (Hôpital d’Instruction des Armées, Clamart, France [PER]) were reported to the National Reference Center for Invasive Mycoses and Antifungals (NRCMA).

Our aim was to clarify the origin/source of infection in both BU1 and BU2. In the absence of genotyping markers for this organism, WGS analysis was performed on 21 isolates (14 from the outbreaks and 7 unrelated) to investigate the links between clinical isolates, understand the epidemiology of the outbreak, and identify and eliminate the potential source of the infections.

## RESULTS

### Clinical and microbiological investigations.

**FIG 1:**
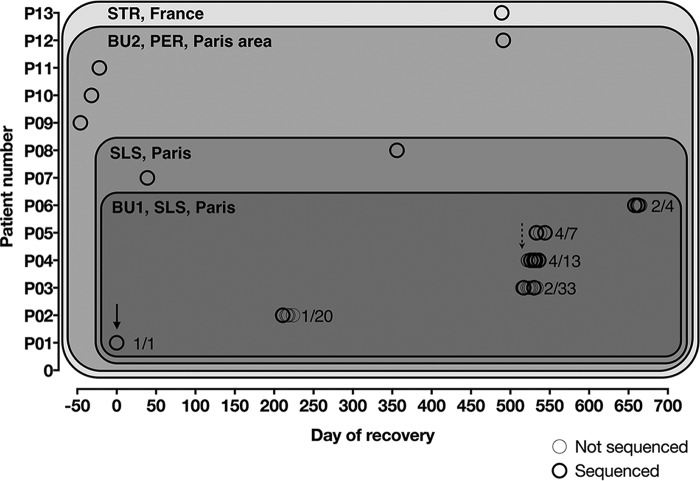
Epidemiological map of 13 patients whose isolates were selected in our study. Isolates from several geographic areas in France (from dark to light gray) have been studied: Burn Unit 1 (BU1) of hospital 1, Paris France; wards of SLS, Paris, France; BU2 of PER in the Paris area and eastern France (one isolate from Strasbourg [STR]). Isolates were prospectively collected for patients P03 to P06. Analyzed isolates are depicted in dark open circles. The index case from BU1 (solid arrow) was thought to be P01, and the outbreak was recognized after P04 got infected (dashed arrow).

Three patients (P03, P04, and P05) developed proven IWM due to M. circinelloides f. circinelloides within 18 days after admission in BU1 (between 18 August and 5 September 2014) and subsequently died from these infections (Table 1). The outbreak was suspected when a positive culture was observed in P04 (11 days after the first positive sample in P03). Sequential samples from the wounds were prospectively obtained starting with P03. A few months later (114 days), another patient (P06) developed proven IWM, and M. circinelloides f. circinelloides was also involved (Fig. 1). We retrospectively noticed isolates from the same species had already been identified in 2013 from two patients in BU1 (P01 with no infection and P02 with proven IWM). A total of seven patients were exposed to (P01 and P07) and/or infected with (P02 to P06) this species in BU1, with P01 as the putative index case. Infection control measures were implemented locally to avoid potential nosocomial transmission to other patients of the unit. More than 30 environmental samples were cultured. All were negative. DNA amplified with the *Mucor*/*Rhizopus* PCR test (22) was detected only in the Bair Hugger filters that were used during the hospitalization of P03, P04, and P05.

**TABLE 1:** Isolates sequenced in this study

Hospital and ward^a^	Isolate ID	Date of recovery (day/mo/yr)	Day from index isolate	Sample type	Patient ID	Site	EORTC/MSG classification	Outcome	Cluster no.	Strain no.^b^
SLS										
BU1	P01_617_BU1_SLS	21/03/13	0	Wound	P01	Wound	No	Alive	C3	S12
	P02_783_BU1_SLS	18/10/13	211	Bone	P02	Wound	Proven	Death	C1	S5
	P03_592_BU1_SLS	20/08/14	517	Wound (shoulder)	P03	Wound	Proven	Death	C1	S1
	P03_594_BU1_SLS	2/9/14	530	Wound (hand)	P03	Wound	Proven	Death	C1	S1
	P04_559_BU1_SLS	29/08/14	526	Wound (hand)	P04	Wound	Proven	Death	C2	S9
	P04_601_BU1_SLS	2/9/14	530	Wound (shoulder)	P04	Wound	Proven	Death	C2	S9
	P04_602_BU1_SLS	4/9/14	532	Wound (forearm)	P04	Wound	Proven	Death	C1	S7
	P04_603_BU1_SLS	8/9/14	536	Wound (leg)	P04	Wound	Proven	Death	C1	S7
	P05_599_BU1_SLS	5/9/14	533	Wound (arm 1)	P05	Wound	Proven	Death	C1	S1
	P05_598_BU1_SLS	5/9/14	533	Wound (thigh)	P05	Wound	Proven	Death	C1	S8
	P05_600_BU1_SLS	5/9/14	533	Wound (arm 2)	P05	Wound	Proven	Death	C1	S8
	P05_622_BU1_SLS	16/09/14	544	Wound (forearm)	P05	Wound	Proven	Death	C1	S3
	P06_023_BU1_SLS	8/1/15	658	Wound (leg)	P06	Wound	Proven	Alive	C1	S6
	P06_032_BU1_SLS	12/01/15	662	Wound (knee)	P06	Wound	Proven	Alive	C1	S6
Pneumology	P07_621_SLS	29/04/13	39	Sputum	P07	Lung	No		C4	S14
Hematology^c^	E01_615_SLS	12/03/14	356	Surface swab					C2	S10

PER										
BU2	P08_701_BU2_PER	3/2/13	−46	Wound	P08	Wound	Proven	Death	C3	S13
	P09_704_BU2_PER	17/02/13	−32	Wound	P09	Wound	Proven	Death	C1	S2
	P10_703_BU2_PER	27/02/13	−22	Wound	P010	Wound	Proven	Death	C1	S2
	P11_702_BU2_PER	25/07/14	491	Wound	P011	Wound	Proven	Death	C2	S11

STR										
Kidney transplant	P12_579_STR	23/07/14	489	Kidney biopsy	P012	Kidney	Proven	Death	C1	S4

a SLS, Hôpital Saint-Louis, Paris, France; PER, Hôpital d’Instruction des Armées, Clamart (a Paris suburb); STR, Strasbourg, France; BU1 and -2, Burn Units 1 and 2.

b Based on strain relatedness after WGS analysis.

c Environmental isolate.

To prevent transmission to other patients, we needed to investigate whether these strains were clonal and needed unrelated isolates from other geographic areas. The additional cases corresponded to an outbreak in BU2 (PER) involving four patients with IWM, as well as one case of proven invasive mucormycosis with kidney invasion in a transplant recipient in Strasbourg (STR) about whom the NRCMA was notified.

Overall, the 12 patients (21 clinical isolates) included 10 cases identified in two burn units (Table 1; Fig. 1).

### Phylogenetic analyses of three loci.

**FIG 2:**
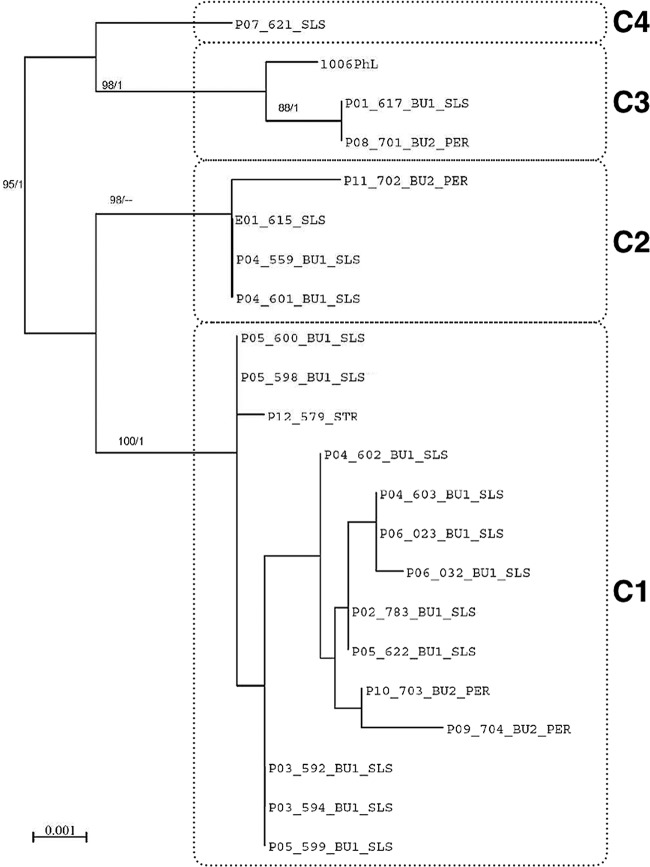
PhyML tree constructed by the maximum likelihood method. The tree was inferred from concatenated 3-locus data set (ITS, 28S, and RPB1). Bootstrap support values from PhyML greater than 70% (left) and a Bayesian posterior probability of >0.80 (right) are shown at the nodes. The 21 clinical isolates are grouped in clades C1 to C4. The scale bar indicates 0.001 nucleotide substitution per character.

Internal transcribed spacer (ITS), D1/D2, and RPB1 sequences were analyzed as separate (data not shown) and combined data sets. The topologies of the multilocus data set by 3 methods (neighbor-joining [NJ], maximum likelihood [ML], and Bayesian inference) were comparable between the individual trees of the three genes analyzed. Four clades (Fig. 2), respectively, denoted C1 (14 isolates, including 11 from BU1), C2 (4 isolates, including 2 from BU1), C3 (2 strains in addition to the reference strain, including 1 from BU1), and C4 (1 isolate), were identified from the analysis of the combined data set, which yielded a significant support (≥95% bootstrap for NJ and ML; 1.0 for Bayesian inference). Isolates recovered from BU1 were distributed in three clades (C2, C3, and C4). All patients from BU1 had at least one isolate included in C1.

### Whole-genome analysis.

To better resolve the diversity of the strains within the four clades and because no further genotyping methods existed for this organism, whole-genome sequencing was performed. Because the biology and the genetics of this organism are poorly understood, we first checked the reproducibility of the sequencing process and the stability of the genome, to be able to define genetically identical strains.

### Establishing the genetic threshold to determine genetically identical strains.

For the three strains isolated from single-spore colonies (i.e., P05_600_BU1_SLS, P04_603_BU1_SLS, and P03_594_BU1_SLS), two complementary approaches were used to estimate the genetic distance between the parent and single-spore colony: estimates of both the evolutionary distance (defined as the proportion of nucleotide differences: i.e., 0.00035, 0.00044, and 0.00029, respectively) and the number of SNP differences (i.e., 4,731, 5,296, and 4,187, respectively). This information gave us the expected genetic proximity measures between pairs of genomes arising from identical strains and independently sequenced isolates. The largest of the three pairs of measures (i.e., 0.00044 mismatch per nucleotide and 5,296 SNPs) was therefore selected as a cutoff below which two compared isolates were defined as belonging to the same strain.

### Experimental investigation of the potential genetic drift of M. circinelloides f. circinelloides*.*

Experimental investigation of the 1006PhL genome upon iterative subculturing on agar (*n =* 3) and three passages in mice (*n =* 3) revealed no acquisition of SNPs during this process, suggesting that the genome of M. circinelloides f. circinelloides was stable upon iterative passages.

### Whole-genome phylogenetic classification of the 21 clinical isolates.

**FIG 3:**
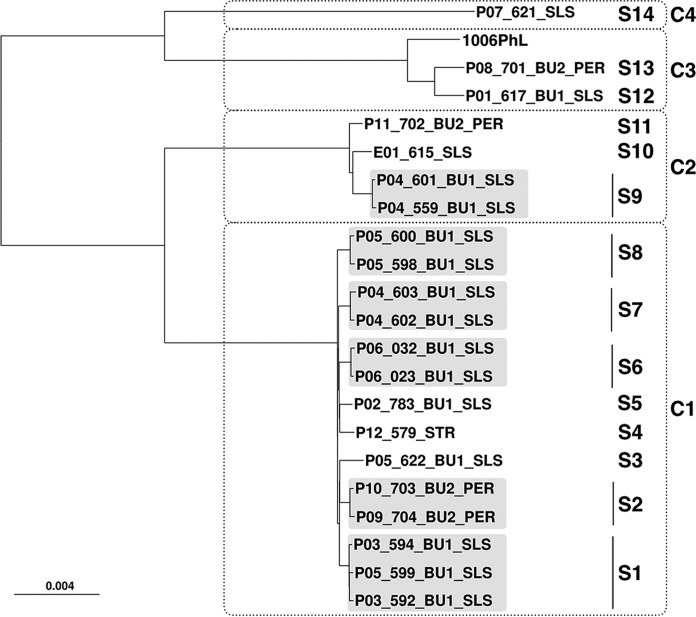
Minimum-evolution phylogenetic tree of the whole genome of 21 clinical isolates and the 1006PhL reference strain. The 21 isolates are grouped in different strains clustered in clades C1 to C4. Clinical isolates belonging to the same strain are highlighted in light gray. The scale bar indicates 0.004 nucleotide substitution per character.

A phylogenetic classification of the whole genome of the 21 isolates was then performed (Fig. 3). This phylogenetic tree allows classification of the genomes in four main clades corresponding exactly to the same clades (C1 to C4) described for the analysis of three loci (Fig. 2). Clades C2, C3, and C4 contained isolates that are clearly distinct from those inside clade C1: e.g., the average estimated evolutionary distances and SNP numbers between isolates from C1 and those inside C2, C3, and C4 are 0.0187, 0.0385, and 0.0390 and 291,467, 702,061, and 715,595, respectively, whereas among C1 isolates, the average pairwise distance and SNP number are 0.00146 and 19,588, respectively (see Table S1 in the supplemental material).

10.1128/mBio.00573-18.1 TABLE S1  Pairwise comparison of the 21 clinical isolates and the 1006PhL reference strain. (Lower left) SNP difference number. (Upper right) Evolutionary distance. Isolate names and associated clade numbers are indicated in the first column and last row. Gray entries indicate values lower than the cutoff (0.00044 mismatch per nucleotide and 5,296 SNPs), below which two compared isolate genomes are considered to belong to the same strain. Download TABLE S1, XLSX file, 0.1 MB. Copyright © 2018 Garcia-Hermoso et al. 2018 Garcia-Hermoso et al. This content is distributed under the terms of the Creative Commons Attribution 4.0 International license.

Furthermore, increased resolution of WGS allowed robust identification of strains (as defined above and in Materials and Methods) and understanding of which clinical isolates belong to which strain. As a result, the 21 investigated isolates and the reference 1006PhL could be partitioned into 14 distinct strains (S1 to S14 [Fig. 3; Table 1]).

### Outbreak dynamics.

In BU1, the isolate of the potential index case P01 (P01_617_BU1_SLS, S12) was different from the isolates subsequently recovered in BU1. The isolate from P02, corresponding to the specific strain S5 clustered in C1 with isolates from P03, P04, P05, and P06. P03 and P06 were also infected with two isolates from S1 (594 and 592) and S6 (032 and 023), respectively. P04 was infected over 10 days with two strains, S9 and S7, belonging to C2 (isolates 601 and 559) and C1 (isolates 602 and 603), respectively. P05 was infected over 11 days with isolates belonging to three strains from C1, S8 (isolates 598 and 600), S3 (isolate 622), and S1 (isolate 599). Indeed, P04 and P05 had mixed infections during the course of their disease, suggesting initial contamination with a mixture of strains, the latter strain also recovered in P03. This suggests cross contamination or common infection in P3 and P5. This has also been observed in BU2, where two patients (P09 and P10) shared strain S2 (isolates 703 and 704), which clustered in C1. Two patients (P11 and P08) were also infected with strains that belonged to C2 and C3, respectively. The environmental (isolate 615) and the colonization (isolate 621) isolates from our hospital clustered in C2 and C4, respectively. The patient from eastern France clustered in C1 (S4), but with a specific strain different from the other C1 strains, as expected for a geographically unrelated infection.

## DISCUSSION

Because isolation of Mucorales is rare in the hospital, the observation of the same species in two independent samples or patients has long been considered a sufficient criterion to suspect and assess transmission or common contamination. Here, we investigated further outbreak-related and -unrelated isolates based on WGS analysis. To our knowledge, this is the first time WGS analysis has been used to resolve an outbreak of invasive Mucorales infection in the context of nosocomial acquisition.

Because WGS was applied for the first time in this setting, we first sought to evaluate reproducibility of the sequencing process and intraculture variation/stability during *in vivo* passage. As the genome of three selected strains was sequenced and assembled twice, we were able to compare contig sets belonging *a priori* to the same strain. This method led to the definition of a pairwise distance cutoff. Therefore, if two genome sequences belonging to different isolates have a pairwise distance below this cutoff, it was inferred *a posteriori* that the two isolates corresponded to the same strain. This cutoff should vary as a function of the organism, the method of sequencing, the bioinformatics pipeline, and the pathophysiology of the disease, suggesting that such data should be obtained each time an investigation of an outbreak due to rare organisms is undertaken. Of note, using pairwise evolutionary distances could be considered as a fast but accurate alternative to the well-known average nucleotide identity (ANI) approach to compare genomes (23, 24) because both were shown to be linearly correlated (25).

Contrary to the initial hypothesis of a single-strain transmission in BU1, we observed that all of the patients from the BU1 outbreak (P01 to P06) were infected by different strains. Surprisingly, our data revealed that each strain was patient specific in BU1, except for S1, suggesting that the outbreak in BU1 was due to multiple strains present in and acquired from a local environmental “reservoir” containing clonally unrelated isolates. This hypothesis is reinforced by two patients (P04 and P05) with IWM coinfected by more than 2 genetically distinct strains. Another hypothesis is that the patients could have been exposed to specific strains or a mixture of strains before arriving in BU1. However, a delay between admission and the first positive culture was 16 days (median), making the hypothesis that exposure occurred in the environment of BU1 more likely. In the settings of severe burns, where invasive fungi do sporulate on the wounds, transmission by air and transmission by the hands of health care workers to other patients are both possible. A major point to emphasize is that only two patients from BU1 (P03 and P05) shared the same strain, S1. This feature has also been observed in BU2, suggesting that transmission between patients is possible. However, this does not rule out the hypothesis of a contamination by the same strain from the environment. Thus, while we cannot entirely exclude that mating and also recombination could occur on the surface of the wound, zygospores of Mucor circinelloides have never been observed in wound tissue and are not known to germinate under any conditions tested thus far *in vitro*. Moreover, the conditions of the host environment (elevated temperature and light) are not optimal for sexual reproduction, and thus it seems unlikely that mating and recombination would be occurring in the setting of infection.

Environmental investigation of the outbreak in BU1 failed to identify the source of infection using culture of multiple samples from the environment, as well as by PCR. DNA amplified with the *Mucor*/*Rhizopus* PCR (22) was detected only in the Bair Hugger filters that were used during the hospitalization of IWM patients P03, P04, and P05. Despite the negative result of this investigation, it is likely that the contamination came from a local source because this has already been described with linens or Elastoplast in burn units (5, 15, 16).

Our findings about the genetic structure of M. circinelloides f. circinelloides are reminiscent of the WGS investigations of the *Apophysomyces* sp. outbreak in Joplin (13) or of the Saprochaete clavata outbreak in France (26), which revealed that several genetic groups can be responsible for infections over the same period of time. In our case, in a given restricted area (BU1), we identified a large diversity of isolates responsible for IWM and were not able to find isolates belonging to unique strains recovered in different places. At the other end of this spectrum, for Apophysomyces trapeziformis several genetically identical isolates were recovered in different places at a distance of several miles (13). In the case of Exserohilum rostratum, all outbreak isolates have closely related genomes, suggesting that a unique strain was responsible for the outbreak (27). These differences could be explained by a completely different pathophysiology of the disease and mode of fungal transmission between these fungal organisms.

This outbreak is illustrative of the importance of the sampling strategy. Repeated sampling is paramount, as is avoiding the assumption that one patient should harbor only one strain. Indeed, in our case two patients were infected by mixtures of strains concomitantly, as already described for cryptococcosis (28). Mixed infections and the impact of genetic heterogeneity of isolates from single patients on the interpretation of transmission chains are an emerging theme in molecular epidemiology in the genomic era (29). Our investigation fully supports the view that multisampling is critical to decipher transmission patterns, especially in the context of outbreaks with such long time frames. Here, multiple sampling was only performed prospectively for four patients (P03, P04, P05, and P06), with one isolate stored and studied for all of the remaining patients. The proportion of mixed infection may thus have been higher than detected here if all of the isolates from all of the patients had been investigated. Based on our experience, to investigate a cluster of wound mucormycosis, we recommend sampling and analyzing several wound lesions and several isolates per patient and analyzing isolates recovered at different times. This will have the potential to find the maximal diversity of the strains if present and have a better view of the outbreak dynamics. In outbreaks involving rare organisms for which the biology and the genetics are mostly unknown, we recommend testing the stability of the genome under different conditions, including *in vivo* models to determine the threshold above which to strains can be delineated.

To prevent potential transmission between patients, all patients with a positive culture were isolated and had a dedicated nurse. To avoid persistence of spores in the environment, after a patient’s death or discharge, rooms should be cleaned by using vaporization of 6% H_2_O_2_. In addition, our management of this outbreak led to the implementation of a twice-weekly screening of serum samples from patients hospitalized in BU1 using Mucorales PCR and by immediate prescription of antifungal treatment when the PCR test was positive (22), in addition to isolation of all culture-positive and/or PCR-positive patients and dedicated nurses to prevent the risk of transmission to other patients. So far, with 2 years of hindsight, no additional cases of M. circinelloides f. circinelloides IWM have been observed. This illustrates how prevention through hygiene together with early diagnosis of mucormycosis could improve patient management and help control dramatic outbreaks in hospital settings among populations at risk.

## MATERIALS AND METHODS

### Isolates and patients.

In order to study the genetic relatedness between the BU1 clinical isolates, the following additional isolates identified as M. circinelloides from other sources were selected (Table 1). (i) The first group of isolates were sequential isolates prospectively collected from the skin lesions of 4 of the patients, as previously described (22). (ii) The next isolates were collected in Hôpital Saint-Louis (SLS) but in another ward (one from the environment and another one colonizing a patient). (iii) The next isolates (*n =* 4) were recovered in another outbreak that involved 4 patients hospitalized in another burn unit (BU2) in the Hopital d’Instruction des Armées, Clamart (PER), located in the suburb of Paris, over the same period of time. These isolates have been sent to the French National Reference Center for Invasive Mycoses and Antifungals (NRCMA). (iv) Finally, one isolate was obtained from invasive mucormycosis recovered from the kidney biopsy specimen of a kidney transplant recipient in Strasbourg (STR) in the eastern part of France.

All positive slants were subcultured once on Sabouraud dextrose agar with gentamicin and chloramphenicol (Bio-Rad, Marnes-la-Coquette, France) at 30°C using the bulk and never single colonies.

Overall, 21 isolates were selected for further analysis: 15 clinical isolates from BU1 (SLS) (12 bulk cultures, including 1 from colonization), 1 environmental isolate from SLS, 4 clinical isolates from BU2 (PER), and 1 clinical isolate from STR.

The sequence of the Mucor circinelloides 1006PhL strain was used as the reference genome (30).

Five of the six patients had proven IWM according to a modified version of the EORTC/MSG criteria (22, 31), and one was considered colonized and was not treated.

### Environmental investigation in BU1.

Extensive environmental sampling was performed, and mycological contamination was investigated by culture methods on Sabouraud agar (Bio-Rad, Marnes-la-Coquette, France) and 2% malt extract incubated at 30 and 37°C. Overall, 30 specimens from nonsterile material, air, surfaces, and aeration machineries, the technical room, Bair Hugger machines, and dedicated nonsterile material were tested. The Bair Hugger machine allows active warming of the patient. It pulses warm air through a plastic pipe into a blanket applied on the patient.

### Polyphasic identification of isolates.

The 21 isolates were sent to the NRCMA, where the purity was verified and identification to the species-level performed using phenotypic and molecular identification on the bulk culture. In detail, microscopic examination was performed on 5- to 7-day-old cultures growth on 2% malt agar at 30°C. Amplification and sequencing of the ITS1-5.8S-ITS2 region and the D1/D2 region of the large subunit (LSU) ribosomal DNA (rDNA) were performed as described previously (32). The amplification of the RPB1 gene (RNA polymerase II largest subunit) was made with primers RPB1Ac and RPB1Cr (33). The PCR products were then sequenced, and the consensus sequences were obtained as previously described (32). Sequences were subjected to pairwise alignments against curated fungal reference databases available at the online MycoBank database (http://www.mycobank.org/).

### Sequence alignment and phylogenetic analysis.

Multilocus sequence alignments of partial sequences of 3 different loci (ITS, 28S, and RPB1) were performed (32, 34).

The sequences were aligned using MAFFT v.7.308 with default settings. Data from each gene were analyzed separately and combined as a concatenated 3-locus data set. For the multilocus data set, neighbor-joining (NJ) phylogenetic trees were constructed using MEGA6 software (35) with the Tamura 3-parameter substitution model and 1,000 bootstrap replicates. The program PhyML v3.0.1 (36) was used to infer maximum likelihood (ML) phylogeny using the TN93 substitution model and 1,000 bootstrap repetitions. Bayesian analysis with default prior of MrBayes v.3.2 (37) was conducted to determine posterior probabilities. Two analyses were done by running 10^6^ generations in four chains, sampling every 100 generations.

### Whole-genome sequencing and assembly.

Because genotyping methods for M. circinelloides f. circinelloides were lacking, WGS was performed to compare isolates. Libraries were constructed using a Nextera XT DNA sample preparation kit (Illumina) and sequenced with an Illumina NextSeq 500 sequencing system with a 2× 150-nucleotide paired-end protocol. Two lanes from these tagged libraries resulted in ~12.6 million read pairs per strain on average. Statistics of assembly for all the isolates are summarized in Table 2.

**TABLE 2:** Statistics of assembly of the strains sequenced in this study

Isolate ID	No. of contigs	No. of bases	*N*_50_	Coverage depth
P01_617_BU1_SLS	8,462	34,880,780	13,803	67×
P02_783_BU1_SLS	4,642	34,558,450	25,365	150×
P03_592_BU1_SLS	5,295	34,215,243	22,844	80×
P03_594_BU1_SLS	4,637	34,551,072	26,027	162×
P04_559_BU1_SLS	5,585	34,509,945	21,800	103×
P04_601_BU1_SLS	4,795	34,726,648	24,551	150×
P04_602_BU1_SLS	5,041	34,155,516	24,450	85×
P04_603_BU1_SLS	5,479	34,215,113	23,173	76×
P05_598_BU1_SLS	5,257	34,139,893	23,651	70×
P05_599_BU1_SLS	5,247	34,181,601	23,820	94×
P05_600_BU1_SLS	5,562	34,319,442	21,377	117×
P05_622_BU1_SLS	5,760	35,087,509	19,734	55×
P06_023_BU1_SLS	4,648	34,540,329	25,831	174×
P06_032_BU1_SLS	4,950	34,519,384	22,633	130×
P07_621_SLS	7,249	34,741,059	15,678	63×
E01_615_SLS	5,446	34,322,336	18,029	68×
P08_701_BU2_PER	6,530	35,386,009	19,860	202×
P09_704_BU2_PER	5,090	34,266,372	19,215	124×
P10_703_BU2_PER	5,081	34,495,044	20,978	162×
P11_702_BU2_PER	4,100	33,816,248	27,537	133×
P12_579_STR	4,757	34,508,712	24,438	168×
1006PHL	4,316	34,938,514	43,860	148×
P03_594_BU1_SLS_CLONE	5,649	34,391,090	17,573	61×
P04_603_BU1_SLS_CLONE	5,849	34,227,008	19,061	74×
P05_600_BU1_SLS_CLONE	5,684	34,478,201	18,116	73×
SEC_INF1	4,162	34,642,463	42,044	77×
SEC_INF3	3,914	34,582,644	43,611	99×
SEC_VEG1	4,293	34,619,149	39,025	52×
SEC_VEG2	3,986	34,622,087	44,282	109×
SEC_VEG3	4,104	34,662,622	43,314	73×

All sequenced reads were clipped and trimmed with AlienTrimmer v.0.4.0 (38), corrected with Musket v.1.1 (39), merged (if needed) with FLASH v.1.2.11 (40), and subjected to a digital normalization procedure with Khmer v.2.0 (41). For each sample, processed reads were finally assembled with SPAdes v.3.10 (42).

### Whole-genome analysis.

For each pair of assembled genomes, an evolutionary distance (proportion of aligned nucleotide differences) was estimated with Mash v.1.0.2 (sketch size = 100,000) (25). For each sample, sequenced reads were also aligned with minimap2 v.2.9 (https://github.com/lh3/minimap2) against the assembled contigs of the reference strain 1006PhL in order to estimate the number of SNPs between each pair of isolates. Both pairwise evolutionary distance and SNP number difference estimates are available in Table S1 in the supplemental material. Finally, the matrix of evolutionary distances was used to infer a minimum-evolution phylogenetic tree with FastME v.2.1.5 (43, 44).

### Reproducibility of the sequencing process.

In a specific experiment dedicated to determine the reproducibility of all parts of the sequencing process (from extraction to sequence analysis), single-spore isolation was performed for 3 isolates recovered from patients P03, P04, and P05 in BU1 (P05_600_BU1_SLS, P04_603_BU1_SLS, and P03_594_BU1_SLS [Table 1]). Spore suspensions were prepared in sterile water with 0.05% Tween 80 and serially diluted, and spores were enumerated to approximately plate five spores per potato dextrose agar (PDA) plate. After 12 h of incubation at 30°C, plates were inspected under a microscope at ×10 magnification, and germinating spores were then transferred to new PDA plates using a stereomicroscope. One colony was thus selected from each isolate for additional sequencing.

### Genome stability experiments.

Genome stability during vegetative growth and host infections was analyzed with the 1006PhL strain. In brief, the strain was grown on PDA for 1 day. The colony was streaked to isolate a single colony, which was then transferred onto a new PDA plate. After 4 days of incubation at 30°C under the light, the spores were collected and subjected to the same procedures up to three passages (VEG1, VEG2, and VEG3 isolates).

For infection passages, spores were suspended in sterile phosphate-buffered saline (PBS). Male 8-week-old BALB/c mice were infected with 10^6^ spores in 200 µl of sterile PBS via tail vein injection. At day 3 postinoculation, the mice were sacrificed, and their brains were collected and placed onto PDA after brain homogenization. After 1 day of incubation, the fungal colony emerging from the brain was streaked to isolate single colonies. One colony was transferred onto PDA and incubated at 30°C under light for 4 days. Spores were then used as the inoculum for the next infection passage, up to three passages (INF1, INF2, and INF3 isolates). Genomic DNAs of these isolates were used to construct libraries with 180-base fragments and 2- to 3-kb jumps, which were sequenced by an Illumina HiSeq 2000 platform at the University of North Carolina High-Throughput Sequencing Facility (HTSF). Data were analyzed using the same pipelines as that implemented for the clinical isolates.

### Ethics statement.

The murine infection experiment was conducted at the Duke University Medical Center in full compliance with all of the guidelines of the Duke University Medical Center Institutional Animal Care and Use Committee (IACUC) and in full compliance with the United States Animal Welfare Act (Public Law 98-198). The Duke University Medical Center IACUC approved all of the vertebrate animal studies under protocol no. A061-12-03. The studies were conducted in the Division of Laboratory Animal Resources (DLAR) facilities, which are accredited by the Association for Assessment and Accreditation of Laboratory Animal Care (AAALAC).

### Data availability.

All FASTQ files were made available on GenBank under accession no. ERS2032288 to ERS2032317 in BioProject PRJEB23686.
